# Monitoring Dust Storms in Iraq Using Satellite Data

**DOI:** 10.3390/s19173687

**Published:** 2019-08-24

**Authors:** Reyadh Albarakat, Venkataraman Lakshmi

**Affiliations:** 1 School of Earth, Ocean and Environment, University of South Carolina, Columbia, SC 29208, USA; 2 Department of Engineering Systems and Environment, University of Virginia, Charlottesville, Charlottesville, VA 22904, USA

**Keywords:** sand and dust storm, MODIS, Aerosol Optical Depth, Iraq, wind speed

## Abstract

Dust storms can suspend large quantities of sand and cause haze in the boundary layer over local and regional scales. Iraq is one of the countries that is often impacted to a large degree by the occurrences of dust storms. The time between June 29 to July 8, 2009 is considered one of the worst dust storm periods of all times and many Iraq is suffered medical problems as a result. We used data from the Moderate Resolution Imaging Spectroradiometer (MODIS). MODIS Surface Reflectance Daily L2G Global 1 km and 500 m data were utilized to calculate the Normalized Difference Dust Index (NDDI). The MYD09GA V006 product was used to monitor, map, and assess the development and spread of dust storms over the arid and semi-arid territories of Iraq. We set thresholds for NDDI to distinguish between water and/or ice cloud and ground features and dust storms. In addition; brightness temperature data (TB) from the Aqua /MODIS thermal band 31 were analyzed to distinguish sand on the land surface from atmospheric dust. We used the MODIS level 2 MYD04 deep blue 550 nm Aerosol Option Depth (AOD) data that maintains accuracy even over bright desert surfaces. We found NDDI values lower than 0.05 represent clouds and water bodies, while NDDI greater than 0.18 correspond to dust storm regions. The threshold of TB of 310.5 K was used to distinguish aerosols from the sand on the ground. Approximately 75% of the territory was covered by a dust storm in 5 July 2009 due to strong and dry northwesterly winds.

## 1. Introduction

Most of the dust storms occur in arid and semi-arid regions and they impact human health, economy, and natural resources [1]. Dust storms generally occur in areas with dry climate where the average annual rainfall of less than 100 mm [2]. Dust storms are formed when strong winds blow over surfaces covered by loose and dry soil and lack vegetation coverage [3,4]. Sand and dust storms (SDS) originate over loose soil or sand and the strong winds pick up this material and causes a significant decrease in visibility. Finer dust particles are entrained in the air by suspension, which is a process that moves soil from one place and deposits it in another [5]. There are many variables that affect the formation of dust storm such as soil moisture, amount of precipitation, drought duration, desertification extent and human activities [3,6]. The propagation of dust storms (SDS) are primarily due to wind over the land surface of arid and semi-arid regions [7]. At low wind speeds, there will be no motion, but when the wind speed reaches the threshold value a number of particles will start to vibrate [7]. The movement of the particles through one of three modes of transport is dependent on particle size, shape and density and fine dust particles are suspended in the air and travel through the wind and are deposited hundreds or even thousands of kilometers away from their source [7]. Previous studies of dust storms have identified sources [8], mechanisms [9,10] and variables impacting the dynamics of this transport [11,12]. Multiple research has suggested that 20% of SDS is formed over Iraq, Saudi Arabia and Iran [13]. Iraq, which is located in between dust storms pathways is characterized with very dry weather during the summer season [14]. Surface wind speed has for the most part been viewed as the fundamental factor that impacts the mobilization of sand and dust particles [15]. Dust events are impacted by wind speed as well as by land surface conditions [16,17,18,19,20]. Long periods of improper cultivation practices, improper use of water resources and environmental changes have contributed to diminished vegetation, desertification, and droughts, and these have contributed to the regional SDS issue. Dry seasons and arid conditions support the disintegration of soil particles, and wind precipitates the formation of SDS [14]. Over this area, dust storms are driven by northwesterly winds, called Shamal (meaning north in Arabic) that travel across central and southern Iraq and pick up dust from the alluvial plains of the Tigris and Euphrates Rivers (http://earthobservatory.nasa.gov/NaturalHazards) [14,21]. 

Shamal generally occurs and is the strongest during the summer months between June and September and decreases during the winter months [21]. In this study, we are focusing on the time period June 29 to July 8, 2009, which is considered the strongest dust storm period in Iraq during the last decade [13,21]. Iranian weather news agency ranked this event as the worst in three decades that the country had experienced [22]. A large dust storm moved through Iraq in the first week of July 2009. As indicated by news reports, Iraqis believed the storm to be the most disastrous in living memory. Several individuals reported to the emergency clinics for respiratory pain, and the dust interfered with air and land travel for nearly a week (https://earthobservatory.nasa.gov/images/39206/dust-storm-over-iraq). The Moderate Resolution Imaging Spectro- radiometer (MODIS)/ Aqua data and ground observations (wind speed and wind direction) have been used to assess the dust storm conditions over Iraq and the surrounding areas (i.e. parts of Saudi Arabia, Kuwait, and Iran). Dust storms have been monitored in the past. However, these past efforts have been limited and earlier research has been based on ground observations and true-color images [6]. We examined the relationship between the wind speed and direction (from the ground meteorological stations) and the MODIS Aerosol Option Depth (AOD), true color images and Brightness Temperature (TB) using satellite remote sensing data. Additionally, this research examines the potential of Normalized Difference Dust Index (NDDI) to monitor SDS, where NDDI is calculated using the following expression [6]:

(1) $$\mathrm{NDDI}=\frac{\left(\rho 2.13\;\mu m-\rho 0.469\;\mu m\right)}{\left(\rho 2.13\;\mu m+\rho 0.469\;\mu m\right)}$$

where *ρ*2.13 μm and *ρ*0.469 μm represent the surface reflectance observations at the top of the atmosphere at 2.13 and 0.469 μm, wavelengths respectively, which correspond to MODIS bands 3 and 7, respectively and these are available at 500 m resolution. Statistical analysis will be used to determine thresholds of NDDI, TB to create maps that show the extraction of dust storm areas on the study area.

## 2. Data and Methods

### 2.1. Data

**Figure 1:**
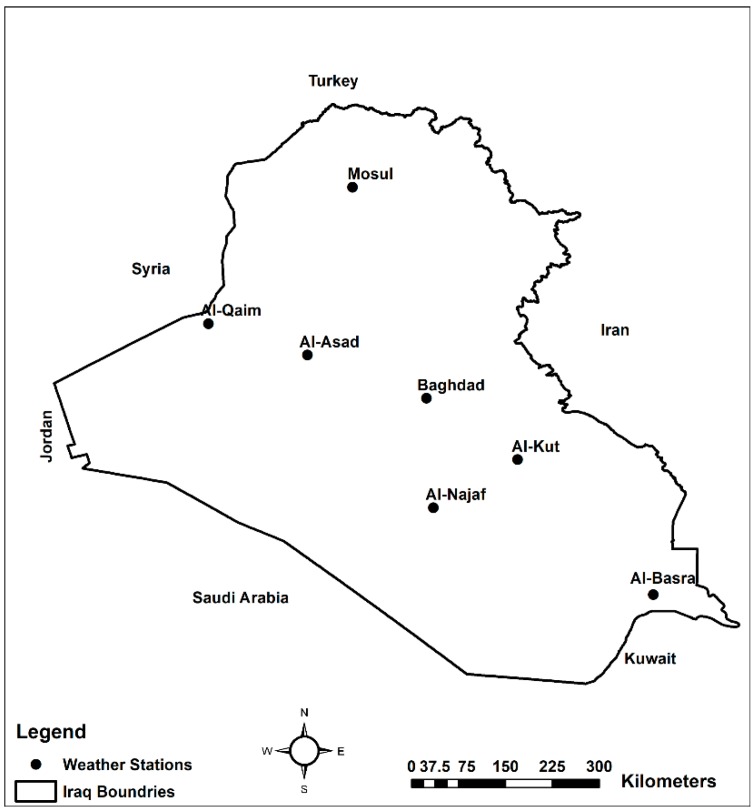
The location of weather stations in the study area.

Daily satellite images from MODIS/Aqua surface reflectance products were used in this analysis. MODIS contains 36 spectral bands, which range from 0.415 μm to 14.235 μm in wavelength. Data are at three different spatial resolutions depending on the spectral band, i.e., 250 m, 500 m and 1 km for spectral bands (1–2), bands (3–7) and bands (8–36), respectively [23]. The specific spectral signatures of different materials cataloged in the Advanced Spaceborne Thermal Emission and Reflection Radiometer (ASTER) spectrum library are utilized as a reference for material determination [24]. The reflectance of water and ice cloud, sand, grass, soil, urban and water in the MODIS bands (0.4–2.5 μm) have been analyzed. In general, the reflectance of dust (sand and soil) increases with wavelength between 0.4 and 2.5 μm with a minimum value in MODIS band 3 (0.469 μm) and a maximum value in MODIS band 7 (2.13 μm) [6]. This study utilized the MODIS/Aqua Surface Reflectance Daily L2G Global V006 products (MYD09GA) to estimate the spectral reflectance for calculating NDDI and to create true-color images by combining bands (1, 4 and 3) respectively. In addition, thermal daily TB data (MYDTBGA) (band 31 centered at 10.78–11.28 μm) was used to separate airborne sand and dust pixels from ground sand and dust [6]. We selected the MODIS level 2 MYD04 deep blue 550 nm AOD data for the period June 29 through July 8, 2009 to confirm the validity of NDDI, the RGB bands and thermal band results. Deep blue AOD data has been extensively validated by multiple researchers in field studies [25]. Additionally, deep blue data preserves accuracy even over bright desert surfaces, while other satellite-based algorithms have difficulty obtaining precise observations [26,27]. Wind speed and wind direction information necessary for validation of satellite-retrieved dust storms were acquired from ground-based weather stations (Figure 1). We selected certain stations (Mosul, Baghdad, Al-Asad, Al-Kut, Al-Qaim, Al-Basra and Al-Najaf) from Iraqi Meteorological Organization and Seismology. The wind speed and direction were measured by anemometer at a height of 10 m above the ground level [28].

### 2.2. Sand and Dust Detection Method

**Figure 2:**
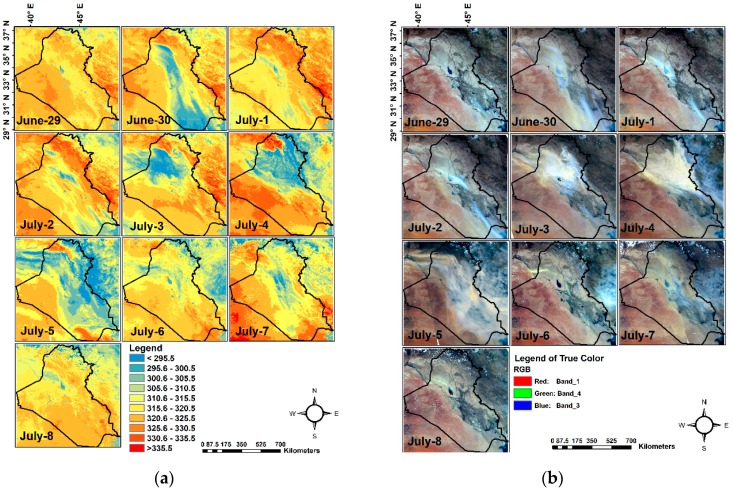
(**a**)TB images of Aqua MODIS band 31 (10.78–11.28 μm) for 10 days (June 29–July 8, 2009) over Iraq and surroundings; (**b**) Aqua MODIS true-color images bands (1, 4 and 3) respectively for 10 days (June 29 to July 8, 2009).

In this research, NDDI was used to map the SDS by using the threshold methodology to separate dust areas from other features i.e. water and ice clouds and ground features except ground sand and dust. Given the high reflectance at band 3 (0.469 μm) and low reflectance at band 7 (2.13 μm), a value of < 0.05 was found to be a reasonable threshold for masking cloud and water bodies [6]. After analyzing all available NDDI images for the study period it was found that the pixels with NDDI values lower than 0.05 represent clouds and water bodies, while NDDI values less than equal to 0.18 for surface features (0.05 < NDDI ≤ 0.18) and higher than 0.18 (NDDI > 0.18) for SDS. We analyzed the TB of Aqua/MODIS band 31 (10.78–11.28 μm) (Figure 2a). TB is used to separate the airborne sand and dust, and the ground sand and dust (aerosol pixels are cooler in contrast to ground sand and dust). Figure 2a illustrates the threshold of TB of 310.5 K that was used to differentiate aerosols from ground sand. TB and NDDI results were compared against the mosaicked true color images built using Aqua/MODIS using bands (1, 4 and 3) (Figure 2b). The determination of the thresholds of NDDI (cloud mask and water bodies) and TB were used for mapping dust storms area in the study. We have calculated the percentage of area covered by dust storms. The percentage was calculated by dividing the area covered via dust storms by the total area of the study area. Table 1 shows the area coverage in km^2^ of the dust storms for the 10 days period between June 29 and July 8, 2009 as well as the percentage of the coverage by dust storms in each day. The covered area was the largest on July 5 and the smallest on June 29. A positive relationship was found between the dust storm area and wind speed, i.e. an increase in wind speed resulted in an increase of the dust storm area. The most significant dust-related activity occurs in the northwestern and western parts of Iraq. These regions are covered by deserts and are characterized by a lack of vegetation during the summer season (June, July, and August) and no rainfall [29]. Figure 2a and Figure 1b showed that the dust emissions in the Tigris and Euphrates alluvial plain reach maximum during the month of July [30]. Shamal winds mobilize the dust and sand particles from Tigris and Euphrates River Basins and carry it towards Western Iran and the Persian Gulf [30]. 

## 3. Results

### 3.1. Relationship between NDDI, TB, and True Color

NDDI computed using MODIS/Aqua surface reflectance data was applied to monitor dust storms formation and spread over Iraq and the surrounding areas. NDDI values lower than 0.05 represent clouds and water bodies, while NDDI values less than equal to 0.18 for surface features (0.05 < NDDI ≤ 0.18) and higher than 0.18 (NDDI > 0.18) for SDS. The 10 categories of TB are outlined in Figure 2a were selected based on Natural breaks (Jenks) algorithm provided by ArcGIS software [31] and are representative of different features observed in the area, i.e. water, clouds, dust, and other ground features. A sensitivity test was carried out to evaluate the feature-specific TB threshold values. It was found that a threshold of 310.5 K allows us to distinguish between dust storms and the ground dust and sand.

**Figure 3:**
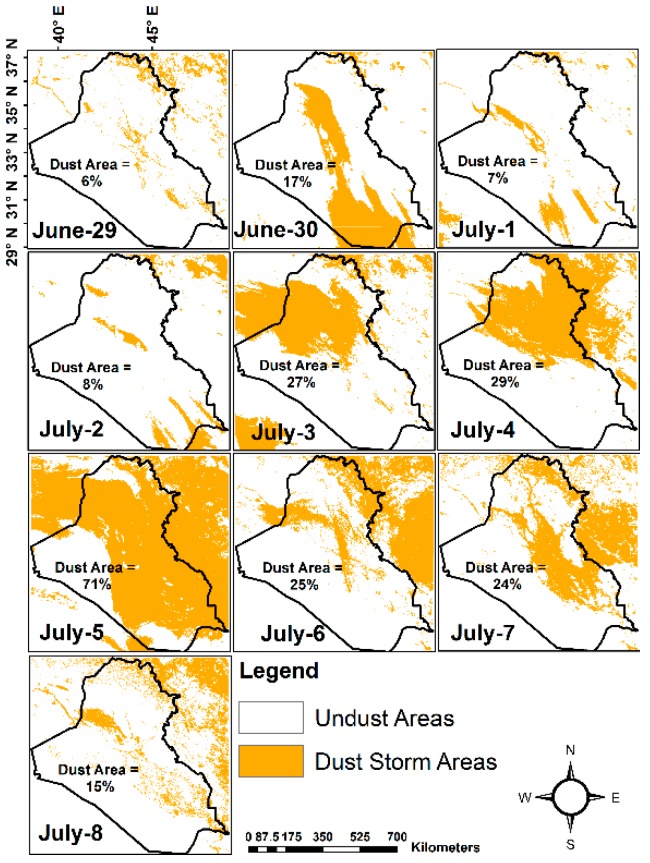
Shows the extraction of the percentage of dust storm areas; clouds, water bodies and other ground features are eliminated.

TB greater than 310.5 K represents ground sand and dust whereas the dust storm regions have TB less than 310.5 K because aerosol and dust pixels are cooler than ground dust and sand [6]. Generally, dust storms occur due to the convective currents that are caused by the heating of the Earth’s surface. The warm air ascends upward in the convective currents, this combined with the relatively cold winds cause fluctuations in the atmospheric pressure and heat. This, in turn, causes dust accumulation and carries sand grains up to a level commensurate with the strength of the wind, drought and soil disintegration [32]. The map of TB extraction of dust storm areas (Figure 3) illustrates the dust storm gradually started to increase around June 30 and reached its maximum coverage and strength on July 5. The dust storm started to decrease after July 6. On July 7, sand and dust blew again over the Central and Eastern parts of Iraq. We also compared the NDDI and TB against true color composites to monitor the dust storms and validate the detection results.

Figure 3 shows the dust storm areas after masking the clouds, water bodies, and ground features (dust and sand particles). As discussed previously, July 5 is associated with the largest areal coverage of dust storm over the region. The dust storm percentages area on June 29, 30 and July 1 to 8, as compared to the total selected area were 6, 17, 7, 8, 27, 29, 71, 25, 24 and 15% respectively, as summarized in Table 1. Approximately, three quarters of the area was covered by dust storm in July 5 due to a strong and dry Shamal winds. The West of Iraq, Western Tigris and Euphrates alluvial plain and Eastern part of Syria are more active areas of dust emission during selected days.

### 3.2. Aerosol Optical Depth (AOD) and Wind Speed and Direction

**Figure 4:**
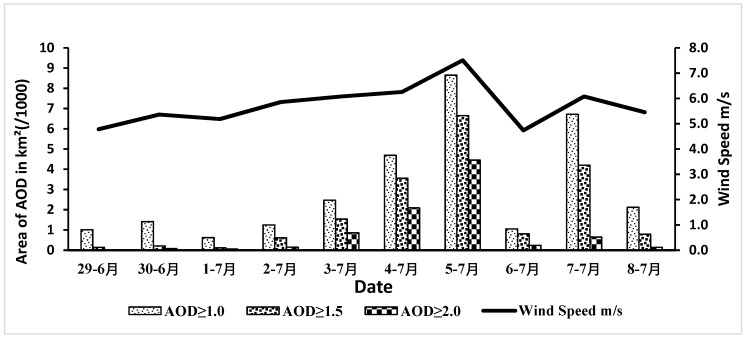
Illustrates the occurrences of AOD for three classes (AOD ≥ 1.0, AOD ≥ 1.5 and AOD ≥ 2.0) with wind speeds through 10 days (June 29–July 8, 2009).

AOD is the degree to which aerosols prevent the transmission of light by absorption or scattering of light [26]. AOD is the amount of aerosol in the atmosphere. In this study, we used MODIS deep blue 550 nm AOD because deep blue data is able to provide aerosol properties even over bright arid land surfaces [27]. However, Figure 4 shows areas of AOD in matching with the wind speed during the 10 days study period. The AOD values were used to classify the dust-outbreak states using classifications from the previous research of Park et al. [33]. According to Park et al. [33], AOD values from 1.0 to 3.0 represent the dust-outbreak state. In our research the AOD was classified into three classes (AOD ≥ 1.0, AOD ≥ 1.5 and AOD ≥ 2.0). High correlation coefficient values (0.8691, 0.8853 and 0.8844) between wind speeds and the three classes of AOD ≥ 0.1, ≥ 1.5 and ≥ 2, respectively. Additionally, strong wind speeds and dust storm area were found to have high correlation (0.825). This indicates that there is a matching of satellite data with ground observations. The strongest wind speed occurred on July 5 when it reached about 8 m/s.

The occurrence of the total number of AOD for three classes reached a maximum on July-5 According to Iraqi news agency, hundreds of Iraqi people reported to the hospital because of blowing dust in the first week of July 2009 [34]. Annually, the Shamal winds blow from mid-June through September and reach the speed on 8 m/s (speed required to launch the dust off the ground) [35]. Shamal winds can continue for several days, and can create destructive dust storms [14]. However, the wind speed sensors at the weather stations (Figure 1) are installed at a height of 10 m above the ground. Weather stations in Iraq measure wind speed and wind direction. The most common, included in complete weather stations, is the anemometer, which typically consists of a rotating vane to measure the direction and a shaft with cups attached that spins with the wind to measure its speed.

**Figure 5:**
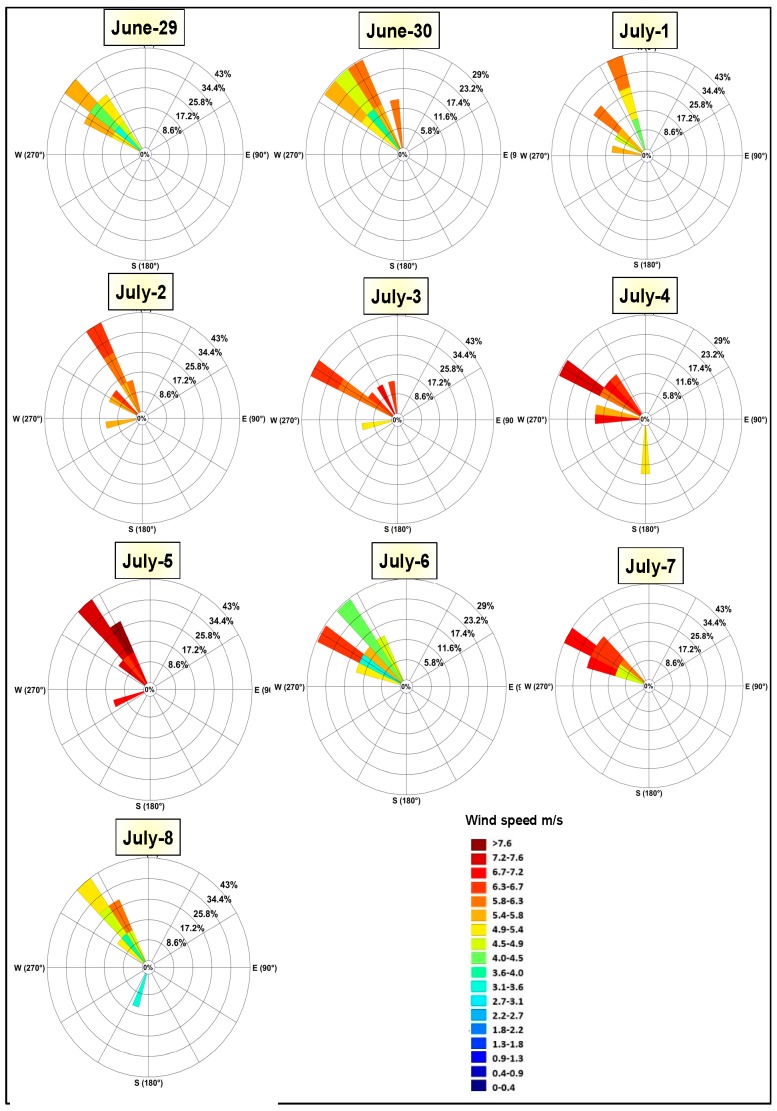
Wind rose plot for the measurement sites for 10 days (June 29–July 8, 2009).

Figure 5 shows the continuous northern wind for ten days all the weather station locations (Figure 1). Wind speed and land surface conditions such as soil moisture are the main factors that led to the worst dust storm over Iraq. A major drought occurred in 2009 that caused a decrease in runoff from the two main rivers in Iraq (Tigris and Euphrates). This resulted in reduction of the water flow and vegetation coverage in the Mesopotamian marshlands [36,37]. Additionally, high temperature and negligible amount of precipitation between June and September of the year in Iraq increased the number of dust storms over the study region [14,38]. Frequently, temperature during summer season exceeds 45 °C [39]. The SDSs over Iraq and surroundings have severe effects on the human health, transportation, and other societal activities. The intensity of aerosol burdens during the period mentioned above was a result of the combined effect of the seasonal drying of the multiple lakes and marshes, the resulting SDS lasted for several days [34].

## 4. Discussion and Conclusions

A combination of satellite data from MODIS/Aqua and meteorological data (wind) were used to monitor and analyze the occurrence of dust storms between June 29 and July 8, 2009 in Iraq. Comparing NDDI, TB, true color images and AOD data showed reasonable agreement and match with ground-based observations of wind speed and wind direction. NDDI values lower than 0.05 represent clouds and water bodies, while NDDI values less than equal to 0.18 for surface features (0.05 < NDDI ≤ 0.18) and higher than 0.18 (NDDI > 0.18) for SDS. The AOD was classified into 3 categories (AOD ≥ 1, AOD ≥ 1.5 and AOD ≥ 2), they showed high correlation values (0.8691, 0.8853 and 0.8844) respectively with wind speeds. The threshold of TB was 310.5 K that used to distinguish aerosols from ground sand. Pixels have greater than 310.5K represent aerosols but ground sand particles were represented by pixels that show TB values more 310.5 K. Approximately, seventy percent of the territory was covered by dust storm in July 5 due to strong and dry northwesterly winds. This research showed good correspondence between satellite detection and ground meteorological data (wind speeds and directions). It is seen that both NDDI and TB have distinctive threshold values over Iraq in contrast with the previous studies. Qu et al. [6] calculated the NDDI to detect SDS over the Gobi region. They used the negative value of NDDI (NDDI < 0.0) for clouds, less than equal to 0.28 for surface features (0.0 < NDDI ≤ 0.28) and higher than 0.28 (NDDI > 0.28) for dust and sand particles. They presented TB threshold of 275 K to recognize the airborne from ground sand and dust. Li and Song [40] applied the NDDI and they identified threshold value of 0.26 to identify sand and dust storms. Butt et al. [41] used negative NDDI values to determine clouds. Additionally, they identified the NDDI values less than 0.23 for surface features, and greater than equal 0.23 (NDDI ≥ 0.23) for SDS. Butt et al. [41] found the TB threshold value of 290 K to distinguish the SDS, and ground sand and dust. Xie et al. [42] demonstrated an enormous contrast in the NDDI and Bright Temperature Difference (BTD) (12, 11) values among cloud and dust. cloud had negative BTD (12, 11) and NDDI values, while in contrast, dust had positive values of those two indices. The reason for this difference in the threshold values of NDDI and TB is due to the difference in the environment, which in turn affects the temperature of SDS, air, and sand on the surface of the earth. However, as discussed, the main reasons that led to the worst dust storms that swept over Iraq in July specifically in summer 2009 were lack of rain, high temperature in the neighboring countries and mismanagement of water and lack of irrigation of agricultural lands [43]. All these factors created the drought in the summer of the year 2009 led to dust storms [43,44,45]. Additionally, the strong Northwesterly Shamal winds promote erosion from the alluvial deposits in the Tigris and Euphrates basin. Al-Dousari et al. [46] identified eight major SDS trajectories, one of them was The Mesopotamian Flood Plain (The Mesopotamian marshes) that due to the marshlands having been subjected to many drastic conditions over a long time [43].

The above study provides a framework for using readily available satellite data to carry out post-analysis of the dust storms. Such an analysis will help decision-makers to send relief aid to the affected communities and to mobilize services for loss of agriculture, industry, or other livelihood. This work fits in with many other works carried out by many others. There has been seminal work done by Doronzo et al. [47] as a part of a special section on the study of dust storms. They outlined the fact that dust travels long distances – the dust from Sahara can reach the Amazonia; the dust from North Africa affected Europe in 2011 and these and others were topics that were discussed in an international conference on dust. A more recent work by Doronzo et al. [48] has showcased research on movement of dust storms in the dry environments in a special issue. They show that dust impacts various areas, cover different parts of the world and the science of movement of dust is well-advanced. The work by Middleton and Kang, [49] analyses the impact of dust storms and outlines methods to minimize impacts from these storms in a global fashion. Some of the impacts listed in their work include soil loss, crop root exposure, air pollution, disruption of transport, disease, salinization, and reduction of solar potential to name a few. They outline methods to control the dust production using vegetation and fencing. Al-Hemoud et al. [50] study the impact of dust storms on oil and gas industry in Kuwait and assess the loss in revenue in drilling, project management and above all encroachment of sand on exploration structures resulting in malfunctioning of equipment. Kim et al. [51] show that planting vegetation (trees and shrubs) decreased the particulate matter due to dust storms in the environment in Seoul, South Korea. Hamdan et al. [52] characterize the fine particulate matter in the atmosphere using laboratory techniques of x-ray fluorescence, scanning electron microscope and x-ray diffraction. The identified the major chemical and mineral compositions of the particulates. Al-Dousari et al. [46] identified eight major trajectories of the major dust storms that originate in the Middle East region (and classified according to size of particles) using remote sensing from NOAA AVHRR, MODIS and TOMS (Total Ozone Mapping Spectrometer) as well as on-site sampling using dust traps and laboratory techniques similar to the ones mentioned above. Cao et al. [53] use meteorological data to study dust storm events and generate a sand dust storm index at several locations all across China and specifically in the Tree-North Forest Shelterbelt Program region and identified the origin of the sand at these locations.

We are using satellite sensors to detect dust storms that have a profound impact in many countries of the Middle East and elsewhere where the dust storms would travel. With the advent of CubeSats and SmallSats, it is quite possible in the near future to have dedicated satellite sensors some even in geostationary orbits with channels similar to the MODIS channels so that we can monitor dust storms in specific geographic areas and issue advance warnings with sufficient lead times. This will help protect life and property. This study definitely presents one particular application of the MODIS sensor. Past missions have not been directed to this particular application and it is our hope that this work will make the audience who are attuned to satellite sensor development aware of such important societal applications.

Our analysis is the first step in identifying communities that get repeatedly impacted by dust storms. Such an analysis will help land-use managers to control development in such areas (including mitigation measures) so as to avoid harm to life and property. We can develop a robust methodology to provide early warnings to the population and provide a systematic approach to the prediction of dust storms—one of the most common natural hazards in this region.

## Figures and Tables

**Table 1:** Dust Storm Areas for June-29–July-8 2009.

Date	Dust Area in Km^2^	Percentage of Dust Storm Area (%)
Jun-29	52,381.3	6
Jun-30	141,593.4	17
Jul-1	51,425.7	7
Jul-2	57,533.8	8
Jul-3	234,754.1	27
Jul-4	251,102.8	29
Jul-5	540,640.8	71
Jul-6	209,680.6	25
Jul-7	206,160.8	24
Jul-8	128,364.8	15
